# Supplementary material for: A Genome of Temperate Enterococcus Bacteriophage Placed in a Space of Pooled Viral Dark Matter Sequences
Source: Viruses. 2023 Jan 12;15(1):216. doi: 10.3390/v15010216 (PMC9865981; doi:10.3390/v15010216)
Supplement: Supplementary file 1 [file viruses-15-00216-s001.zip › Supplementary Materials.pdf]

Pchelin IM, Tkachev PV, Azarov DV et al. A genome of temperate *Enterococcus* bacteriophage placed in a space of pooled viral dark matter sequences

Supplementary Materials

Table S1. Host range of Enterococcus phage VEsP-1.

	Strain number	Origin	Species	VEsP-1 lytic activity	EOP value
1	GT15	Probiotic strain	*Bifidobacterium longum*	absent	0.0
2	CCUG 18657	Public collection strain	*Enterococcus casseliflavus*	absent	0.0
3	m26	Intestine of woolly mammoth corpus	*Enterococcus durans*	absent	0.0
4	232	Clinical isolate	*Enterococcus faecalis*	absent	0.0
5	258	Clinical isolate	*Enterococcus faecalis*	absent	0.0
6	262	Clinical isolate	*Enterococcus faecalis*	absent	0.0
7	288	Clinical isolate	*Enterococcus faecalis*	absent	0.0
8	296	Clinical isolate	*Enterococcus faecalis*	absent	0.0
9	312	Clinical isolate	*Enterococcus faecalis*	absent	0.0
10	326	Clinical isolate	*Enterococcus faecalis*	absent	0.0
11	336	Clinical isolate	*Enterococcus faecalis*	absent	0.0
12	894	Clinical isolate	*Enterococcus faecalis*	absent	0.0
13	24988	Clinical isolate	*Enterococcus faecalis*	absent	0.0
14	26555	Clinical isolate	*Enterococcus faecalis*	absent	0.0
15	10 arctic	Ornitogenic soil, Svalbard	*Enterococcus faecalis*	absent	0.0
16	114-1	Ornitogenic soil, Svalbard	*Enterococcus faecalis*	absent	0.0
17	147-1	Ornitogenic soil, Svalbard	*Enterococcus faecalis*	absent	0.0
18	159-1	Ornitogenic soil, Svalbard	*Enterococcus faecalis*	absent	0.0
19	164-1	Ornitogenic soil, Svalbard	*Enterococcus faecalis*	absent	0.0
20	167-1	Ornitogenic soil, Svalbard	*Enterococcus faecalis*	absent	0.0
21	174-1	Ornitogenic soil, Svalbard	*Enterococcus faecalis*	absent	0.0
22	184-1a	Ornitogenic soil, Svalbard	*Enterococcus faecalis*	absent	0.0
23	186-1	Ornitogenic soil, Svalbard	*Enterococcus faecalis*	absent	0.0
24	244-1	Ornitogenic soil, Svalbard	*Enterococcus faecalis*	absent	0.0
25	33-2	Ornitogenic soil, Svalbard	*Enterococcus faecalis*	absent	0.0
26	369-1	Ornitogenic soil, Svalbard	*Enterococcus faecalis*	absent	0.0
27	453-2	Ornitogenic soil, Svalbard	*Enterococcus faecalis*	absent	0.0
28	459-1	Ornitogenic soil, Svalbard	*Enterococcus faecalis*	absent	0.0
29	5 arctic	Ornitogenic soil, Svalbard	*Enterococcus faecalis*	absent	0.0
30	520-2	Ornitogenic soil, Svalbard	*Enterococcus faecalis*	absent	0.0
31	69-1	Ornitogenic soil, Svalbard	*Enterococcus faecalis*	present	0.1
32	75(2.3)	Ornitogenic soil, Svalbard	*Enterococcus faecalis*	absent	0.0
33	78-1	Ornitogenic soil, Svalbard	*Enterococcus faecalis*	absent	0.0
34	84-1	Ornitogenic soil, Svalbard	*Enterococcus faecalis*	absent	0.0
35	ATCC 29212	Public collection strain	*Enterococcus faecalis*	absent	0.0
36	CCUS 52538	Public collection strain	*Enterococcus faecalis*	absent	0.0
37	Nik	Clinical isolate	*Enterococcus faecalis*	present	1.2

38	Serg	Clinical isolate	*Enterococcus faecalis*	present	1.0
39	13c	Clinical isolate	*Enterococcus faecalis*	present	0.6
40	Sh	Clinical isolate	*Enterococcus faecalis*	absent	0.0
41	160	Clinical isolate	*Enterococcus faecium*	absent	0.0
42	237	Clinical isolate	*Enterococcus faecium*	absent	0.0
43	276	Clinical isolate	*Enterococcus faecium*	absent	0.0
44	280	Clinical isolate	*Enterococcus faecium*	absent	0.0
45	334	Clinical isolate	*Enterococcus faecium*	absent	0.0
46	344	Clinical isolate	*Enterococcus faecium*	absent	0.0
47	578	Clinical isolate	*Enterococcus faecium*	absent	0.0
48	872	Clinical isolate	*Enterococcus faecium*	absent	0.0
49	1074	Clinical isolate	*Enterococcus faecium*	absent	0.0
50	2018	Clinical isolate	*Enterococcus faecium*	absent	0.0
51	2184	Clinical isolate	*Enterococcus faecium*	absent	0.0
52	25559	Clinical isolate	*Enterococcus faecium*	absent	0.0
53	26588	Clinical isolate	*Enterococcus faecium*	absent	0.0
54	27228	Clinical isolate	*Enterococcus faecium*	absent	0.0
55	27249	Clinical isolate	*Enterococcus faecium*	absent	0.0
56	176a	Ornitogenic soil, Svalbard	*Enterococcus faecium*	absent	0.0
57	206-1	Ornitogenic soil, Svalbard	*Enterococcus faecium*	absent	0.0
58	321v	Clinical isolate	*Enterococcus faecium*	absent	0.0
59	52-3	Ornitogenic soil, Svalbard	*Enterococcus faecium*	absent	0.0
60	CCUG 52539	Public collection strain	*Enterococcus faecium*	absent	0.0
61	l2-faecium	Ornitogenic soil, Svalbard	*Enterococcus faecium*	absent	0.0
62	L3	Probiotic strain	*Enterococcus faecium*	absent	0.0
63	m38	Putative ancient strain	*Enterococcus faecium*	absent	0.0
64	CCUG 18658	Public collection strain	*Enterococcus gallinarum*	absent	0.0
65	K1	Healthy individual isolate	*Enterococcus gallinarum*	absent	0.0
66	72a	Healthy individual isolate	*Enterococcus hirae*	absent	0.0
67	DH5α	P. Clearly collection strain. USA	*Escherichia coli*	absent	0.0
68	30151	Clinical isolate	*Staphylococcus aureus*	absent	0.0
69	SA774	Clinical isolate	*Staphylococcus aureus*	absent	0.0
70	SA775	Clinical isolate	*Staphylococcus aureus*	absent	0.0
71	SA776	Clinical isolate	*Staphylococcus aureus*	absent	0.0
72	SA778	Clinical isolate	*Staphylococcus aureus*	absent	0.0
73	47167	Clinical isolate	*Staphylococcus epidermitidis*	absent	0.0
74	55-13	Clinical isolate	*Streptococcus agalactiae*	absent	0.0
75	79-13	Clinical isolate	*Streptococcus agalactiae*	absent	0.0
76	97-13	Clinical isolate	*Streptococcus agalactiae*	absent	0.0
77	B31	Clinical isolate	*Streptococcus pyogenes*	absent	0.0
78	V31	Clinical isolate	*Streptococcus pyogenes*	absent	0.0
79	V32	Clinical isolate	*Streptococcus pyogenes*	absent	0.0
80	V53	Clinical isolate	*Streptococcus pyogenes*	absent	0.0

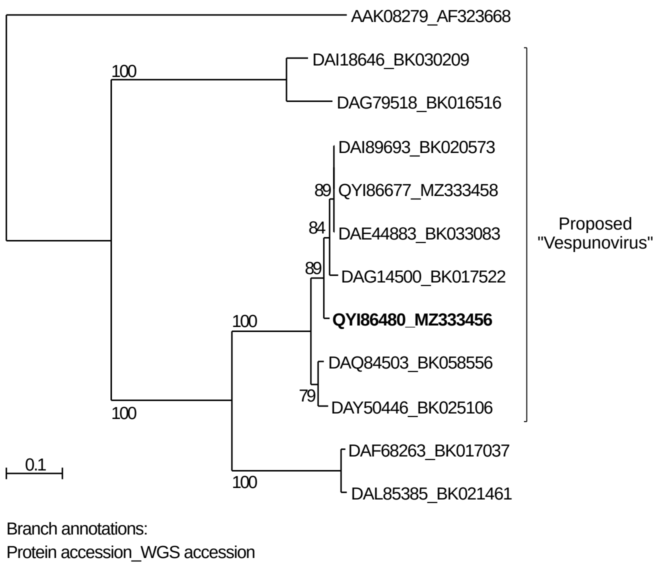

Figure S1. Maximum Likelihood phylogenetic tree inferred from tail protein sequences. The amino acid sequence sample was obtained by querying the NCBI Protein database with VEsP-1 tail protein QYI86480.1 by a blastp search. The tree was calculated with the use of PhyML 3.1 program [Guindon et al. 2010] as implemented in SeaView 5.0.4 package [Gouy et al. 2010]. The substitution model was LG, assuming 12 across site rate variation categories. The number of invariable sites was algorithm-optimized. The stability of branches was assessed with 100 bootstrap replicates calculated by the Transfer Bootstrap Expectation method [Lemoine et al. 2018]. Branch support values above 75% are shown. The VEsP-1 sequence is highlighted in bold. The topology of the tree does not corroborate the “Vespunovirus” hypothesis.

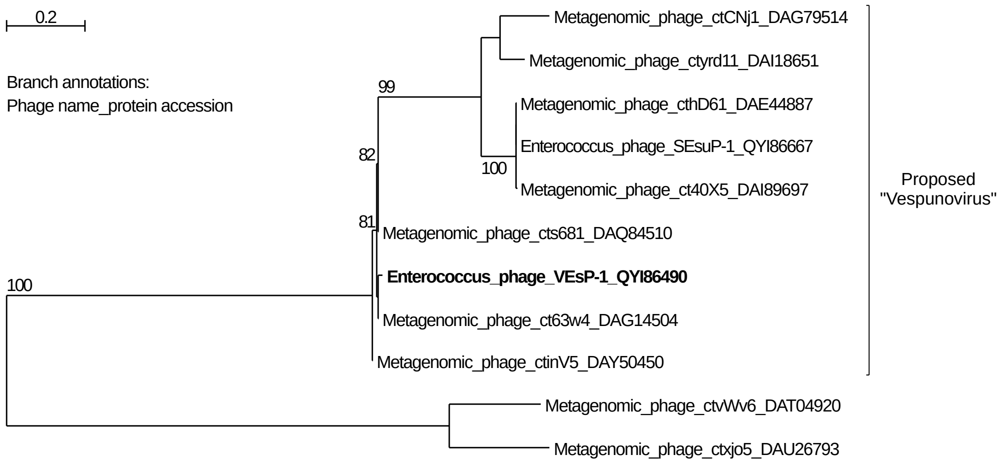

Figure S2. Maximum Likelihood phylogenetic tree inferred from major capsid protein sequences. The amino acid sequence sample was obtained by querying NCBI Protein database with VEsP-1 major capsid protein QYI86490.1 in a blastp search. The tree was calculated as described in the caption of Figure S1. The topology of the tree supports the “Vespunovirus” hypothesis.
